# 1816. Bloodstream Infection Following Arterial Aneurysm Repair: A Population-Based Study

**DOI:** 10.1093/ofid/ofac492.1446

**Published:** 2022-12-15

**Authors:** Hussam Tabaja, Supavit Chesdachai, Larry M Baddour, Randall DeMartino, Brian D Lahr, Daniel C DeSimone

**Affiliations:** Mayo Clinic, Rochester, Minnesota; Mayo Clinic, Rochester, Minnesota; Mayo Clinic Rochester, Rochester, Minnesota; Mayo Clinic, Rochester, Minnesota; Mayo Clinic Rochester, Rochester, Minnesota; Mayo Clinic, Rochester, Minnesota

## Abstract

**Background:**

Bloodstream infection (BSI) following arterial aneurysm repair may signify vascular graft infection (VGI). The incidence of BSI and its implications in patients with arterial grafts have received scant attention with limited contemporary study. Therefore, the aim of the current investigation is to describe the incidence and epidemiology of BSI following arterial aneurysm repair in a population-based cohort.

**Methods:**

Retrospective case-cohort study using the expanded Rochester Epidemiology Project (e-REP) to identify all arterial aneurysm repairs performed in adults (>18 years) residing in eight counties in southern Minnesota between January 1, 2010 and December 31, 2020. All repairs were performed at Mayo Clinic Rochester. Electronic health records were screened for initial BSI episode following aneurysm repair and complicating VGI.

**Results:**

Overall, 643 patients underwent 708 aneurysm repairs: 417 endovascular and 291 open surgical repairs. Forty-two patients developed BSI during 2,992 cumulative person-years of follow-up. The median time from repair to BSI was 3.1 [interquartile range 0.5, 6.4] years. The BSI incidence rate, in cases per 1000 person-years, was 14.0 (95% CI 10.1-19.0) overall, including 18.2 (95% CI 11.9-26.7) following open surgical repair and 10.2 (95% CI 5.8-16.6) following endovascular repair (p=0.148). Thirty-nine (92.9%) of the BSI cases were monomicrobial, with 18 (46.2%) due to Gram-positive organisms and 21 (53.8%) due to Gram-negative organisms. Of the 42 patients with BSI, 10 (23.8%) had VGI. VGI occurred in 8/18 (44.4%) monomicrobial Gram-positive BSI as compared to 2/21 (9.5%) monomicrobial Gram-negative BSI (p=0.013). The 3-year mortality rate following BSI was 52.3%.

**Conclusion:**

There was no statistically significant difference in incidence rates of BSI following open surgical repair and endovascular repair. Higher rates of VGI were seen with Gram-positive BSI as compared to Gram-negative BSI.

**Disclosures:**

**Larry M. Baddour, M.D.**, Boston Scientific: Advisor/Consultant|Botanix Pharmaceuticals: Advisor/Consultant|Roivant Sciences: Advisor/Consultant|UpToDate, Inc.: Royalty payments - authorship duties.

